# Supplementary material for: MS2DeepScore: a novel deep learning similarity measure to compare tandem mass spectra
Source: J Cheminform. 2021 Oct 29;13:84. doi: 10.1186/s13321-021-00558-4 (PMC8556919; doi:10.1186/s13321-021-00558-4)
Supplement: Supplementary file 1 — Additional file 1. MS2DeepScore: a novel deep learning similarity measure to compare tandem mass spectra. Figure S1. Score comparisons with respect to precision-recall in highly unbalanced dataset. Figure S2–S8. Hyperparameter search. Figure S9 Early stopping. Figure S10 Assessing prediction uncertainty using Monte-Carlo Dropout. Table S1. Measuring the linear correlation between actual and predicted scores. Figure S11 t-SNE using MS2DeepScore or modified cosine score. [file 13321_2021_558_MOESM1_ESM.pdf]

Additional file 1

MS2DeepScore: a novel deep learning similarity measure to compare tandem mass spectra

Florian Huber[a*], Sven van der Burg[a], Justin J.J. van der Hooft[b], Lars Ridder[a]

[a] *Netherlands eScience Center, 1098 XG Amsterdam, the Netherlands*

[b] *Bioinformatics Group, Wageningen University, 6708 PB Wageningen, the Netherlands.*

[*] *corresponding author: florian.huber@hs-duesseldorf.de*

This document contains additional figures and experiments and is structured as follows:

Contents

Figure S1 - Score comparisons with respect to precision-recall in highly unbalanced dataset 2

Figure S2 - S8: Hyperparameter search 4

Spectrum binning 4

Network depth 6

Network width 8

Figure S9: Early stopping 10

Figure S10: Assessing prediction uncertainty using Monte-Carlo Dropout 11

Table S1: Measuring the linear correlation between actual and predicted scores 12

Figure S11: t-SNE using MS2DeepScore or modified cosine score 13

Figure S1 - Score comparisons with respect to precision-recall in highly unbalanced dataset

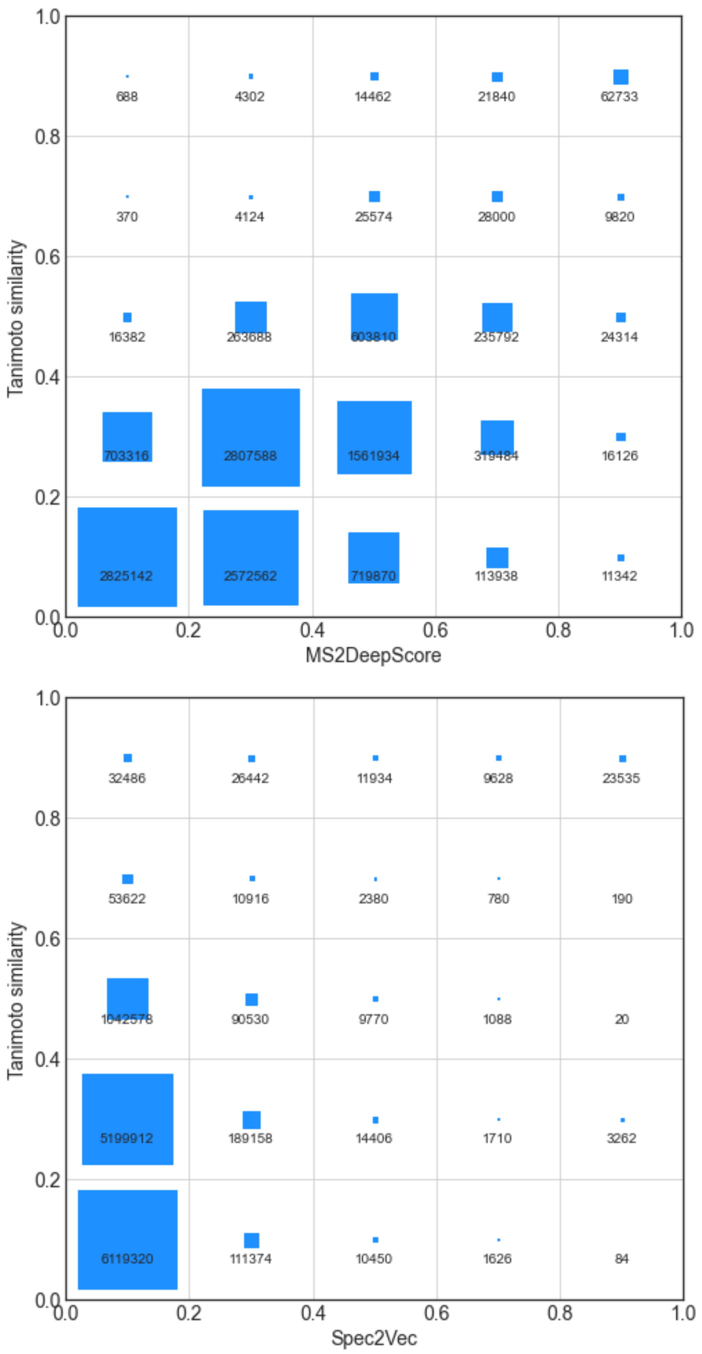

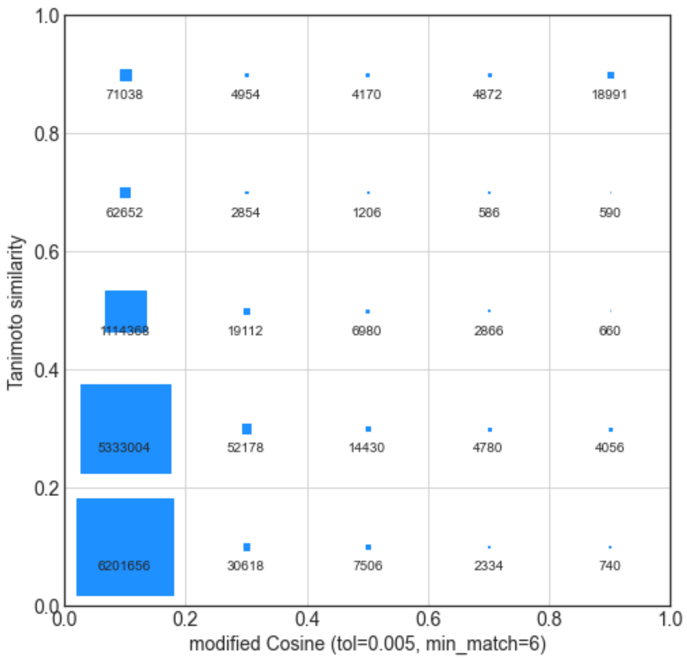

Figure S1. Similar to a confusion matrix for evaluating classification tasks, this plot displays the total fraction of all scores (3601 x 3601 possible pairs) which falls into one of 5 Tanimoto scores bins and one of 5 predicted Tanimoto score bins. The area of the squares reflects the total amount which is also given as absolute numbers.

Figure S2 - S8: Hyperparameter search

Spectrum binning

To be able to handle the sparse MS/MS spectra, we only keep spectrum peaks within m/z values between 10.0 and 1000.0 Da and apply a binning step to reduce the possible number of m/z positions to a desired value. There is an obvious trade-off between more peaks which better reflect peak m/z positions and less bins which drastically reduces the necessary network parameters and computational performance. We were, however, surprised to observe a rather small impact of the spectrum binning on the overall Tanimoto predictions as measured using the root mean squared error (RMSE, Figure S2, top and Figure S3, left), the mean absolute error (MAE, Figure S2, bottom and Figure S3, right). Here, we only varied the input dimension from 500 to 15,000 bins and kept the other network training parameters constant.

The fact that the initial binning has a rather moderate influence on the prediction accuracies gives a first hint at what information is key for the neural network to make a Tanimoto score prediction. Apparently, the larger pattern of the main peaks (i.e. relative positions and intensities) is far more important than the precise m/z locations. For very low numbers of bins (<1000) the prediction accuracy worsens notably and the predicted Tanimoto scores can hardly discriminate nuances in the range between 0.6 and 1.0 (see figure S4). Accuracy keeps improving when binning is becoming finer, but naturally this also results in a rapidly increasing number of model parameters, we decided to keep the input dimension at 10,000 bins.

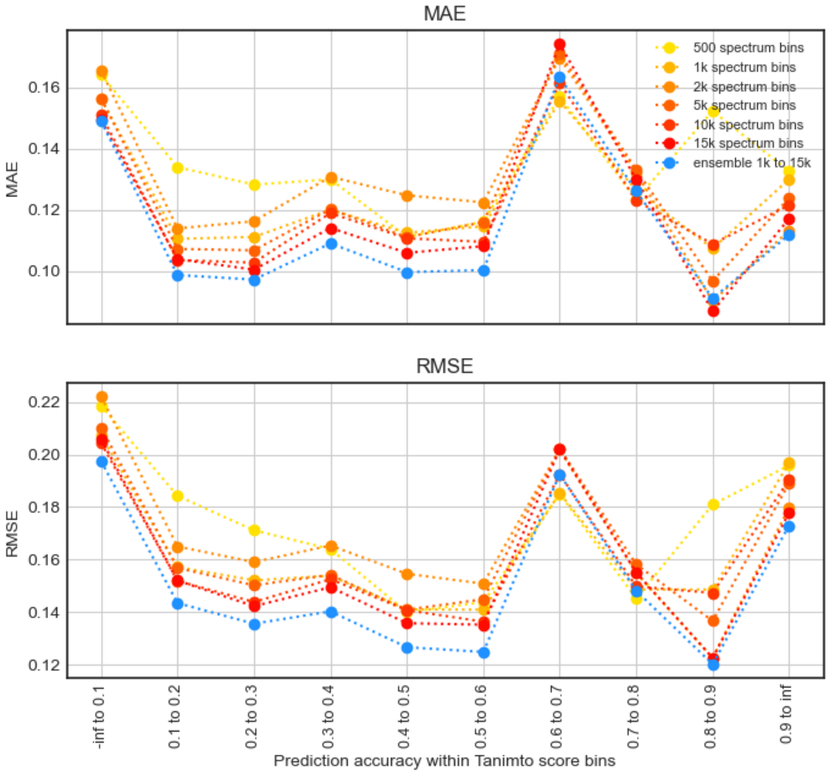

Figure S2. Different MS2DeepScore models were trained for different spectrum compression ranging from 500 to 15,000 bins (considering peaks in the m/z range from 10.0 to 1000.0 Da). In addition, an naive ensemble model was tested which takes the median prediction of the 5 models between 1k and 15k bins, blue dots). The MAEs and RMSEs are here calculated for all spectrum pairs within the validation set (3597 spectra) which fall into one of the 10 possible Tanimoto score bins (x-axis labels).

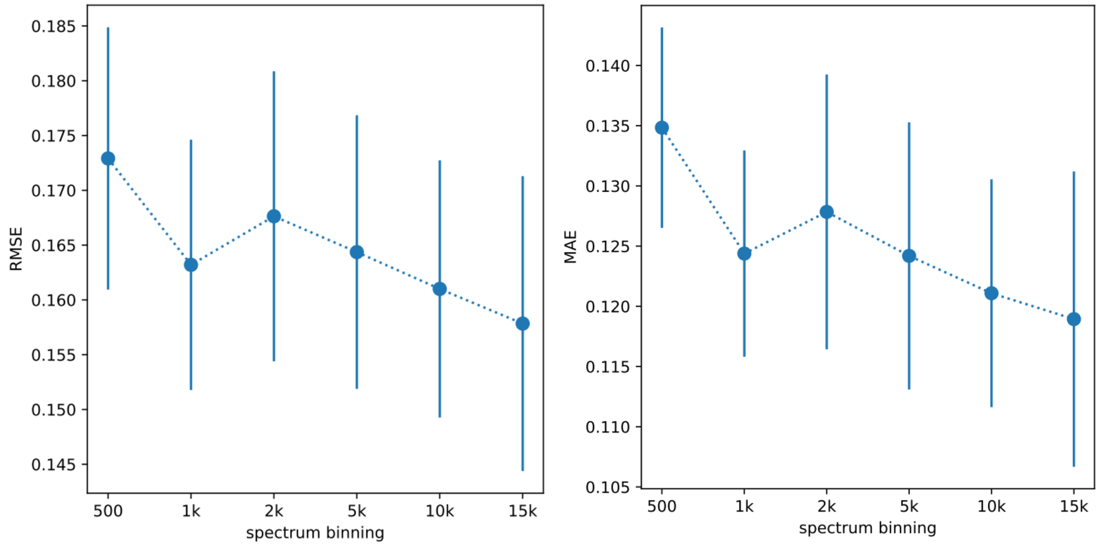

Figure S3. Spectrum were binned to values between 500 to 15,000 bins while all other network training parameters were kept constant. Models were training using early stopping with a patience of 5 epochs. Scores were computed for all possible pairs of spectra within the validation set and compared to the actual Tanimoto scores within 10 bins from 0 to 1.0 to account for the unbalanced nature of the dataset (see figure S2). The respective mean values across the 10 bins are plotted here together with the standard deviation (error bars are -0.5 STD to +0.5 STD). (Left plot) Mean RMSEs for different spectrum binning. (Right plot) Mean MAEs for different binning.

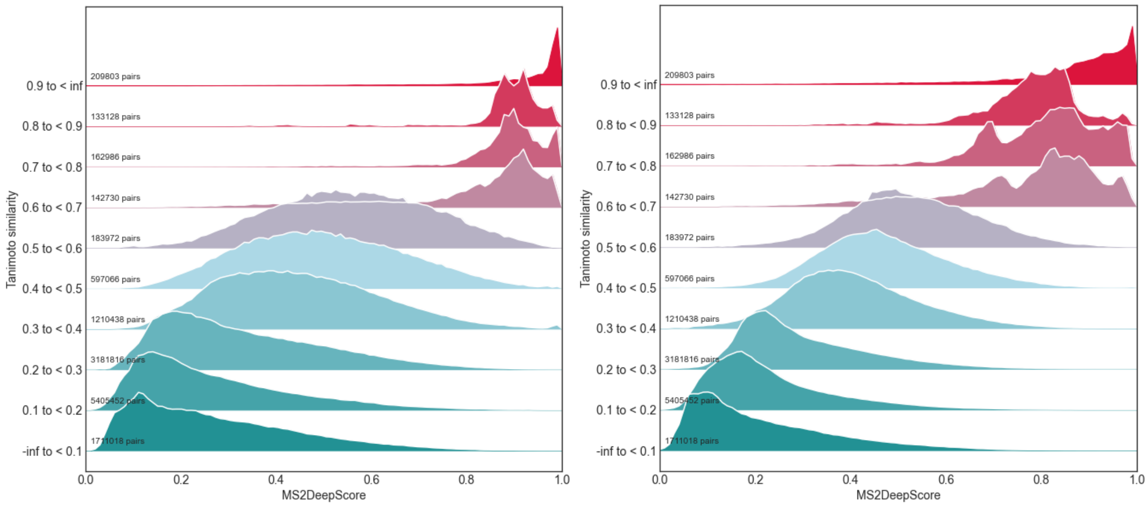

Figure S4. Comparisons between predicted Tanimoto scores (MS2DeepScore) and Tanimoto score (rdkit daylight fingerprint, 2048bits), for 500 spectrum bins (left) and 15,000 spectrum bins (right).

Network depth

Different numbers of densely connected layers were tested. The base network contains a first compression step from dim_bins → dim_01 followed by a number of densely connected layers, and finally an embedding creation → dim_embedding. We tested architectures ranging from 2 layers (10k→ 500→ 200) to 5 layers (10k→ 500 → 500 → 500 → 500 → 200) without achieving any notable improvement when using more than three layers (figures S5 and S6).

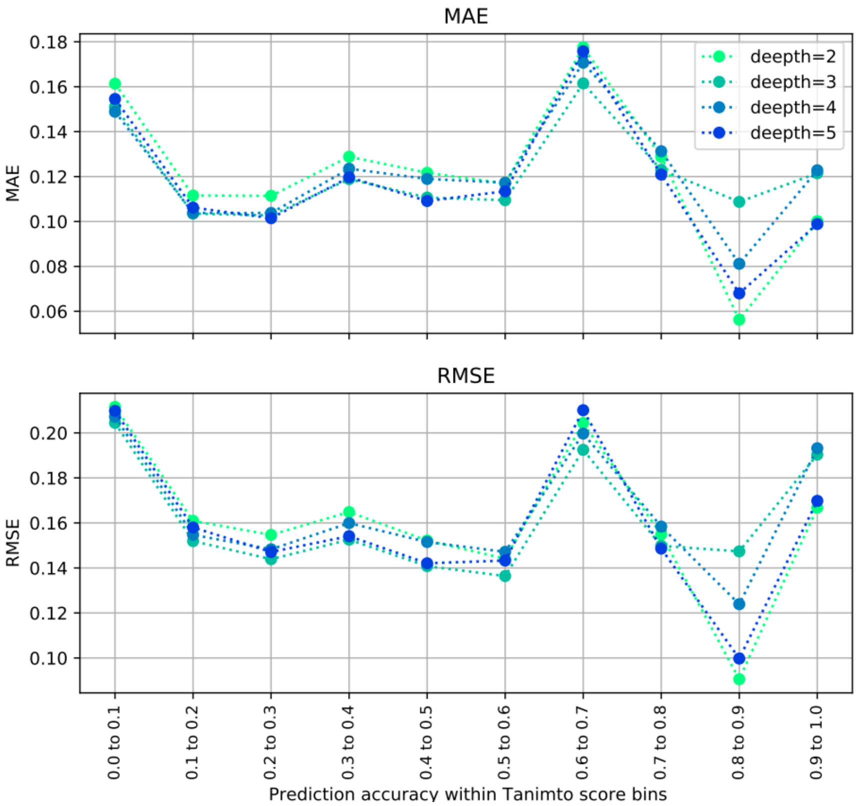

Figure S5. Different MS2DeepScore models were trained for different network depths, ranging from 2 to 5 dense layers. The MAEs (upper plot) and RMSEs (lower plot) are here calculated for all spectrum pairs within the validation set (3597 spectra) which fall into one of the 10 possible Tanimoto score bins (x-axis labels).

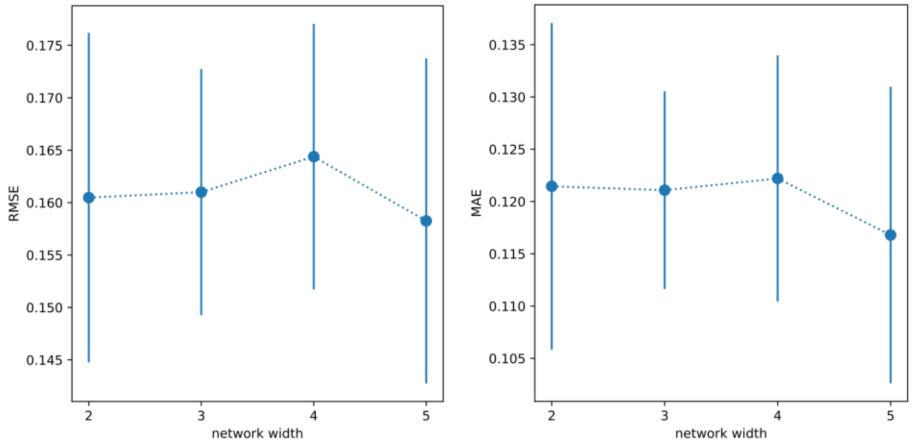

Figure S6. Plot showing the mean values for the RMSEs (left) and MAEs (right) for MS2DeepScore models with 2 to 5 dense layers. Means are simply the means across all 10 Tanimoto score bins shown in figure (figure S5). Error bars range from -0.5STD to +0.5STD based on the RMSE for all 10 Tanimoto bins.

Network width

Analog to the network depth we also tested different network widths, i.e. different number of nodes per dense layer. Mostly due to the large number of weights for the first compression step, the total number of parameters is increasing rapidly for wider networks.

The prediction error generally increases for less nodes, but only really breaks down for very small networks (figure S7, e.g. 10k→ 20→ 20→ 200). It also seems to saturate for a width of about >1000 nodes. To us, this suggests that the key information is extracted in the first network layer where the sparse input is converted to a meaningful representation.

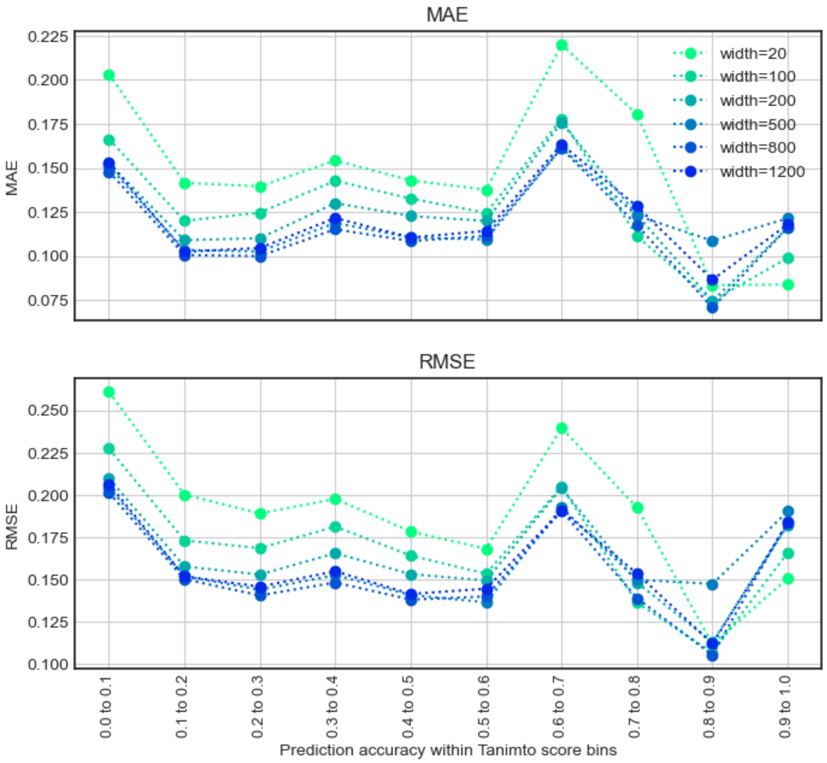

Figure S7. Different MS2DeepScore models were trained for different main dense layer widths ranging from 20 to 1200 nodes. The MAEs (upper plot) and RMSEs (lower plot) are here calculated for all spectrum pairs within the validation set (3597 spectra) which fall into one of the 10 possible Tanimoto score bins (x-axis labels).

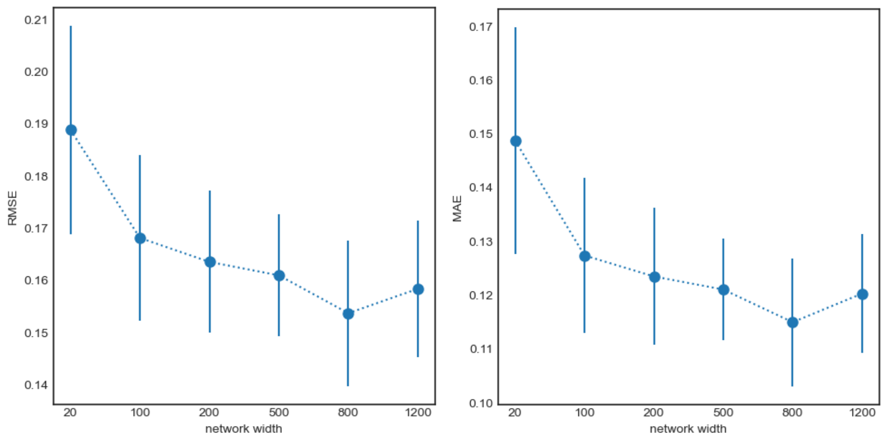

Figure S8. Plot showing the mean values for the RMSEs (left) and MAEs (right) for MS2DeepScore models of central network widths between 20 and 1200 nodes. Means are simply the means across all 10 Tanimoto score bins shown in figure (figure S7). Error bars range from -0.5STD to +0.5STD based on the RMSE for all 10 Tanimoto bins.

Figure S9: Early stopping

We applied early stopping during model training to avoid overfitting. Model training was stopped once the loss (mean squared error, MSE) on the validation set did not improve for 5 epochs in a row.

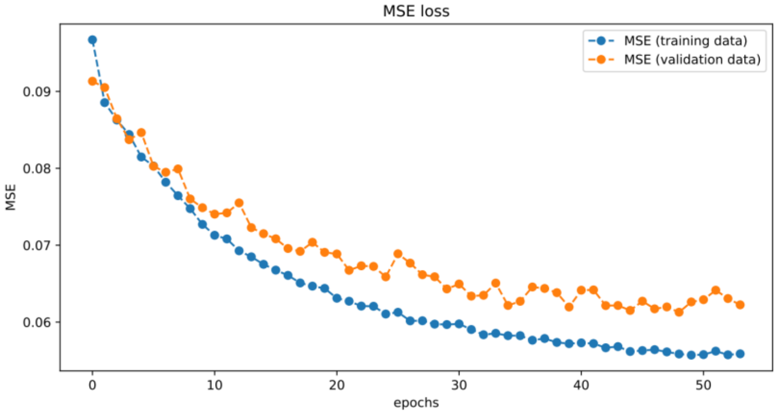

Figure S9. Training and validation loss ('MSE') during training of the model.

Figure S10: Assessing prediction uncertainty using Monte-Carlo Dropout

For assessing the prediction uncertainty of our Siamese neural networks, we implemented Monte-Carlo Dropout. This gives an ensemble of predictions for any input pair of spectra from which we can compute the median similarity score as well as the interquartile range (IQR) as a proxy of the prediction uncertainty. In figure 7 of the main article, the value of this measure of uncertainty is demonstrated by the increased prediction accuracy in a subset of the results with low IQR values.
Figure S10 displays how the IQR values are distributed for different Tanimoto scores (figure S10 A) or predicted Tanimoto scores (figure S10 B). Panel A reveals that the distribution of IQR values is largest for the predictions for compound pairs with actual high Tanimoto scores (>0.9), which agrees with the observation we made in the main manuscript that the prediction error is highest for those scores (figure 7C, main manuscript). Panel B of the figure indicates that the assessed model uncertainty is lowest for very high (>0.9) and very low (<0.1) predicted Tanimoto scores. This is contrary to our finding that the observed prediction accuracy for high Tanimoto scores is actually lower than for the rest. We expect that results can further be improved by adjusting the IQR thresholds to account for the shifted IQR distributions.

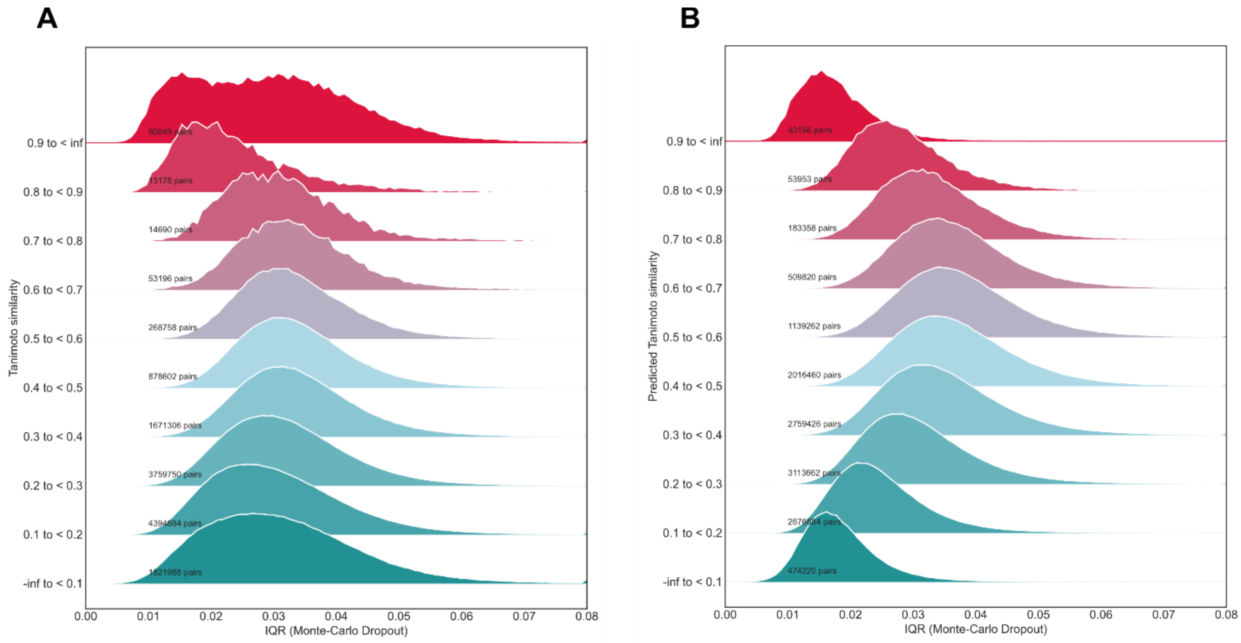

Figure S10. Running Monte-Carlo Dropout on the test set to compute median scores together with the interquartile range (IQR) to assess the model uncertainty. (A) Distributions of IQR values for different Tanimoto score bins. (B) Distributions of IQR values for different predicted Tanimoto score bins. The latter indicates that the prediction uncertainty is (often wrongly) estimated to be lower for the smallest and highest predicted Tanimoto scores.

Table S1: Measuring the linear correlation between actual and predicted scores

In the main manuscript we evaluated the prediction errors by computing the root mean squared errors (RMSE), also for different Tanimoto score bins. An alternative way to evaluate the quality of the predicted similarities can be to measure the linear correlation between predicted and actual scores, for instance using the Pearson correlation coefficient.

Since we generally were mostly interested in a good predictions for higher Tanimoto scores we compared the correlation coefficients for different fractions of the highest similarity scores in the test set. This was done for the four different reference metrics displayed in figure 3 (main manuscript) which were obtained from different molecular fingerprints (Daylight, Morgan-2, Morgan-3), and similarity measures (Tanimoto, Dice).

The table below shows computed Pearson correlation coefficients for the different measures and for different selections of scores. While we generally observe high correlation coefficients for the highest similarity score pairs, the coefficient differ notably between circular fingerprints (Morgan-2 and Morgan-3) and Daylight fingerprints. We expect that this is due to the very pronounced differences in the score distributions, see figure 3A of the main article. This can also explain why the correlation for the highest fraction is slightly larger for the circular fingerprints where high structural similarities are spread over a wider range (lowest 98% of all Morgan-3 scores are all <= 0.2222 whereas the lowest 98% of all Daylight scores are all <= 0.5472, see Table S1), while the correlation coefficient over all scores is better for the Daylight fingerprints.

	**Daylight, Tanimoto**	**Morgan-3, Tanimoto**	**Morgan-2, Tanimoto**	**Morgan-2, Dice**
**Top 2 %**	0.520<br>(scores > 0.5472)	0.579<br>(scores > 0.2222)	0.570<br>(scores > 0.2892)	0.518<br>(scores > 0.4486)
**Top 5 %**	0.542<br>(scores > 0.4673)	0.625<br>(scores > 0.1518)	0.629<br>(scores > 0.1979)	0.614<br>(scores > 0.3304)
**Top 50 %**	0.522<br>(scores > 0.2055)	0.447<br>(scores > 0.0698)	0.481<br>(scores > 0.0875)	0.493<br>(scores > 0.1609)
**All**	0.549	0.389	0.428	0.434

Table S1. Pearson correlation coefficients computed on the test set (3601 spectra) for different conditions and score types. For each of the four shown scenarios a separate model was trained to predict the respective structural similarities based on a pair of mass spectra.

Figure S11: t-SNE using MS2DeepScore or modified cosine score

In figure 8 (main manuscript) we show an example use-case for MS2DeepScore beyond simple pair-wise similarity estimation. Using t-SNE on the MS2DeepScore spectral embeddings, the full 3601 spectra of the test set can be assigned 2D coordinates that to some extend reflect their chemical characteristics. Such a plot can in principle be done with any distance measure, hence also using modified cosine scores (figure S11). Distances were computed as 1 – similarity score for both MS2DeepScore and modified cosine scores. 2D coordinates were then computed using t-SNE from sci-kit learn using perplexity=100 and iterations=1000.

While such plots are difficult to compare quantitatively, we found that the plots generated from MS2DeepScore similarities display more concise clusters of same-colored dots (i.e., same class spectra are close(r) together). The plots obtained with the modified cosine scores also show consistent clusters, but also contain larger regions of fully interspersed spectra from numerous different categories. Furthermore, computational scalability might be an additional argument for the use of MS2DeepScore. Using embeddings (which can be pre-computed if needed), the distance calculations are much faster for MS2DeepScore than for modified cosine scores which makes it feasible to run related analyses even on (much) larger datasets.

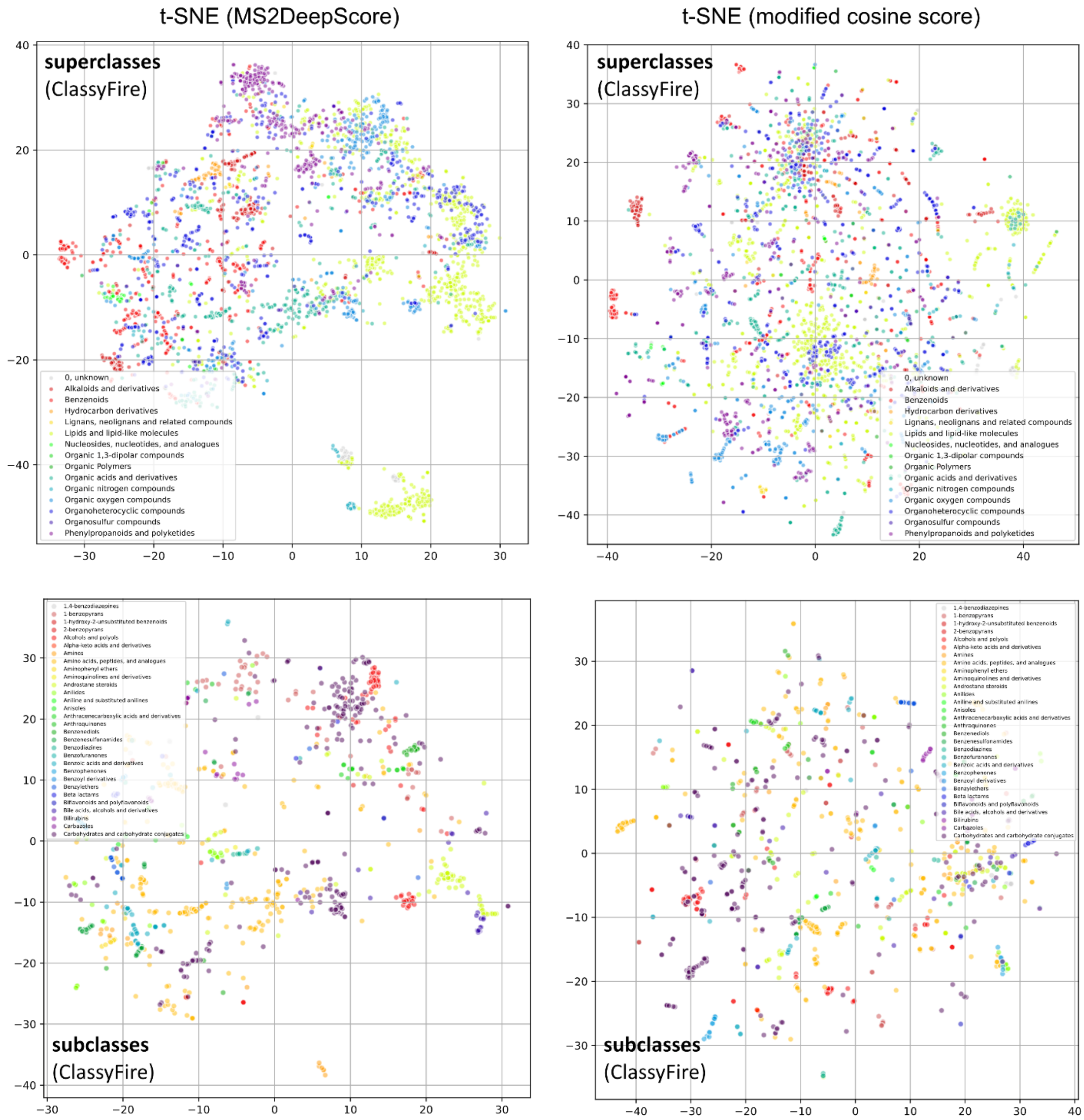

Figure S11. T-SNE plots of the 3601 spectra in the test set. 2D coordinates were computed using t-SNE and distances based on MS2DeepScore (left panel) or modified cosine scores (right panel). Each dot represents one spectrum, and each dot is colored according to its chemical superclass (top row) or subclass (bottom row). For better readability only the first 30 subclasses were plotted. T-SNE was run using a perplexity of 100.
